# 1-Butyl-3-Methylimidazolium Tetrafluoroborate Film as a Highly Selective Sensing Material for Non-Invasive Detection of Acetone Using a Quartz Crystal Microbalance

**DOI:** 10.3390/s17010194

**Published:** 2017-01-20

**Authors:** Wenyan Tao, Peng Lin, Sili Liu, Qingji Xie, Shanming Ke, Xierong Zeng

**Affiliations:** 1 Shenzhen Key Laboratory of Special Functional Materials & Shenzhen Engineering Laboratory for Advance Technology of Ceramics, College of Materials Science and Engineering, Shenzhen University, Shenzhen 510081, China; wenyan_tao@hotmail.com (W.T.); smke@szu.edu.cn (S.K.); 2 Key Laboratory of Optoelectronic Devices and Systems of Ministry of Education and Guangdong Province, College of Optoelectronic Engineering, Shenzhen University, Shenzhen 518060, China; 3 Department of Biomedical Engineering, the Chinese University of Hong Kong, Shatin, Hong Kong 999077, China; sililiu2007@gmail.com; 4 College of Chemistry and Chemical Engineering, Hunan Normal University, Changsha 410082, China; xieqj@hunnu.edu.cn

**Keywords:** 1-butyl-3-methylimidazolium tetrafluoroborate, acetone, quartz crystal microbalance, non-invasive sensing

## Abstract

Breath acetone serves as a biomarker for diabetes. This article reports 1-butyl-3-methylimidazolium tetrafluoroborate ([bmim][BF_4_]), a type of room temperature ionic liquid (RTIL), as a selective sensing material for acetone. The RTIL sensing layer was coated on a quartz crystal microbalance (QCM) for detection. The sensing mechanism is based on a decrease in viscosity and density of the [bmim][BF_4_] film due to the solubilization of acetone leading to a positive frequency shift in the QCM. Acetone was detected with a linear range from 7.05 to 750 ppmv. Sensitivity and limit of detection were found to be 3.49 Hz/ppmv and 5.0 ppmv, respectively. The [bmim][BF_4_]-modified QCM sensor demonstrated anti-interference ability to commonly found volatile organic compounds in breath, e.g., isoprene, 1,2-pentadiene, *d*-limonene, and *dl*-limonene. This technology is useful for applications in non-invasive early diabetic diagnosis.

## 1. Introduction

Human breath contains a large number of volatile organic compounds (VOCs) derived from a metabolic origin [1,2]. Concentration of VOCs is related to the degree of oxidative damage inside the body. VOCs commonly found in exhaled breath include alkanes, alkenes, aldehydes, ketones, sulfides, nitrides, etc. [3]. There are about 340 types of VOCs from a normal individual’s breath. However, VOC composition in breath varies largely from person to person [3]. Breath testing for VOCs is intrinsically safe and noninvasive. Hence, breath VOCs find lots of applications in early disease diagnosis. It has been reported that alveolar-arteriole C_4_–C_20_ alkane gradients were more abundant in patients with lung and breast cancer than those in a normal person [4]. The mean concentrations of pentane and carbon disulfide are significantly higher in the breath of patients with schizophrenia than in a normal person [5]. The nitric oxide level in the exhaled breath of a patient with asthma and community-acquired pneumonia is higher than that in healthy subjects [6,7].

Breath acetone is conventionally used as a biomarker for diabetes. The mechanism, as follows, has been accepted by most people. Type 1 diabetes arises from the loss of insulin-producing β-cells of islets of Langerhans in the pancreas, leading to insulin deficiency. Insufficient insulin can result in hyperglycemia and ketosis. Ketosis is a consequence of an elevated level of lipolysis in adipose tissue and increased ketogenic flux in the liver, resulting in large amounts of ketone bodies, i.e., acetone, acetoacetate, and β-hydroxybutyrate. Type 2 diabetes is characterized by insulin resistance. It is a physiological condition where cells are no longer able to respond to insulin. Cells are not able to take in glucose, amino acids, and fatty acids. It leads to energy deficiency, which brings β-oxidation of fatty acids, producing an excess of acetyl CoA. The resulting excess of acetyl CoA synthesizes ketone bodies, i.e., acetone, acetoacetate, and β-hydroxybutyrate. Acetoacetate can be decarboxylated to volatile acetone.

For type 1 diabetes, the mean values with standard deviation of breath acetone concentrations in different patients are 2.19 ± 1.38 and 5.58 ± 6.68 ppmv [8,9]. For type 2 diabetes, the mean values are 2.05 ± 2.31 and 2.35 ± 0.56 ppmv [9,10]. The mean values for normal subjects are 0.48 ± 0.20 and 0.51 ± 0.18 ppmv in previous reports [9,10]. There is no literature to set a definite cut-off breath acetone concentration between normal and diabetic subjects. It indicated that breath acetone concentration is measured at a higher value in diabetic patients and the concentration is usually below 1 ppmv for healthy individuals. The correlation coefficient between breath acetone and blood sugar has been found to be in the range of 70% to 80% [11,12]. Therefore, we can develop a sensitive method with detectable acetone concentration around 1 ppmv for early diabetes diagnosis. Detection of VOCs in breath is challenging because breath VOCs exist in trace concentrations falling within the range of ppbv and ppmv, thus requiring highly sensitive analytical methods. There are considerable amounts of carbon dioxide and water in the breath. Carbon dioxide may destroy the vacuum environment in the mass spectroscopy system, resulting in unwanted background. Water may interfere with adsorbents in gas chromatography columns. Therefore, a fast, noninvasive, and highly sensitive diagnostic technique is required. Existing noninvasive techniques include photoacoustic spectroscopy [13], cavity ring-down spectroscopy [14], gas chromatography, mass spectrometry [15], and other techniques [16,17]. However, these techniques are labor-intensive, expensive, and difficult to miniaturize in a clinical setting.

Recently, room-temperature ionic liquids (RTILs) have gained much attention in many fields including green chemistry, organic synthesis, gas sensing, and high-conductivity material engineering [18,19]. RTILs are liquids comprised entirely of ions at room temperature. They have unique chemical and physical properties, such as high chemical and thermal stability, ionic conductivity, negligible vapor pressure, low toxicity, and the ability to dissolve a wide range of organic and inorganic compounds [20]. One intrinsic property of RTILs is that they consist only of ions but they can still be made hydrophobic [21]. Their strong affinities for organic species are desirable in the detection of vapors. Owing to their low vapor pressure, there is no loss of RTILs during vaporization. On the other hand, quartz crystal microbalances (QCM) are particularly suitable for sensor fabrication because they are inexpensive, simple to use, and allow the deposition of a wide variety of sensing materials [22,23,24,25]. The working principle is based on the mass-frequency shift relationship for QCM exposure to a sample vapor, which theoretically yields sensitivity in the ppb region. There are reports describing QCM devices based on ionic liquids [26,27,28]. For example, Liang et al. investigated Au-based QCM sensors modified by nine types of RTILs for selected organic vapors. A calibration curve for acetone on a 1-propyl-3-methylimidazolium bis[(trifluoromethyl) sulfonyl]amide-modified sensor was obtained. Thomas et al. performed a feasibility study of artificial olfactory systems using RTILs as selective depositions on Au-based QCM electrode surfaces. Abdul et al. presented the physicochemical interpretation of interaction forces between RTILs and gas vapor on Pt-based QCM sensors. Eight different types of RTILs were used to detect nitromethane and 1-ethyl-2-nitrobenzene. However, testing in a real world setting, such as in the presence of limonene, isoprene, etc., cannot be found.

In this work, we constructed a [bmim][BF_4_]-modified QCM sensor for acetone detection. The sensing principle is based on the strong affinity of acetone with the [bmim][BF_4_] film, which decreases the viscosity and density of the film, and results in a shift in resonance frequency. The piezoelectric quartz crystal impedance (PQCI) method was used to measure the frequency shift, which was found to correlate with acetone concentration.

## 2. Materials and Methods

### 2.1. Chemicals

Isoprene (99.5%) and *d*-limonene (95%) were purchased from Sigma-Aldrich (Shanghai, China). Acetonitrile (99.5%) and acetone (99.98%) were ordered from Scharlau (Barcelona, Spian) and Fisher Scientific Company (London, UK). 1,2-pentadiene (95%) and *dl*-limonene (99.5%) were from Tokyo Chemical Co. (Tokyo, Japan). All chemicals were used without further purification. 1-butyl-3-methylimidazolium tetrafluoroborate ([bmim][BF_4_], (98%)), with water content below 500 ppmv [29], was purchased from Sigma-Aldrich and it was further purified through rotary evaporation under vacuum.

### 2.2. Preparation of the [bmim][BF_4_]-Modified QCM Sensor

9 MHz *AT*-cut quartz crystals (Chen Jing Electronics, Beijing, China) were used as the QCM sensor in this work. The diameter of the quartz crystal was 12.5 mm, and the gold electrode diameter was 6.0 mm. The quartz crystal was fixed to a glass tube using silicone rubber, and only one side of the crystal was coated with film for sensing. Then, 10 µL of [bmim][BF_4_] in 10% acetonitrile was dropped onto the center of the QCM device and it was then loaded on a spin coater (Laurell Technology, North Wales, PA, USA). It was rotated at a speed of 500 rpm for 30 s, and 2000 rpm for 2 min. The film was then blown and dried with N_2_. Excess RTILs on the edge of the quartz wafer were wiped off by acetonitrile soaked cotton. The acetonitrile impurity introduced in this process was removed by purging with dry N_2_. Quartz crystal could be recovered by rinsing with acetonitrile.

### 2.3. Sample Handling Setup and Quantification of Acetone Vapor Concentration

Static mode was adopted for the sample handling system. The modified QCM was placed in a custom-made sealed glass chamber of 273 mL. A fixed amount of pure acetone solution was injected into the chamber, followed by its natural evaporation. The acetone sample is measured when the chamber is full of acetone vapor. The acetone concentration (*C_a_*) in the cell was calculated by:

(1) $$C_{a}=\frac{V_{a}\rho_{a}}{V_{cell}}$$

where *ρ_a_* and *V_a_* are the relative density and sample volume of acetone. *V_cell_* is the chamber volume, which is 35 mL as designed in this work. The relative density of acetone is 0.79 g/mL.

Without loss of generality, the acetone vapor concentration is converted into parts per million (ppm) in this work. The conversion formula is:

(2) $$C_{ppm}=\frac{C_{a}}{\left(\frac{M_{a}}{22.4}\right)*\left(\frac{273}{273+T}\right)*\left(\frac{P}{101325}\right)}$$

where *M_a_* is the molar mass of acetone, *T* is the chamber temperature, and *P* is pressure. Under room temperature (22 °C) and normal atmospheric pressure conditions, Equation (2) can be simplified to:

(3) $$C_{ppm}=0.41C_{a}$$

### 2.4. Measurements

**Scheme 1:**
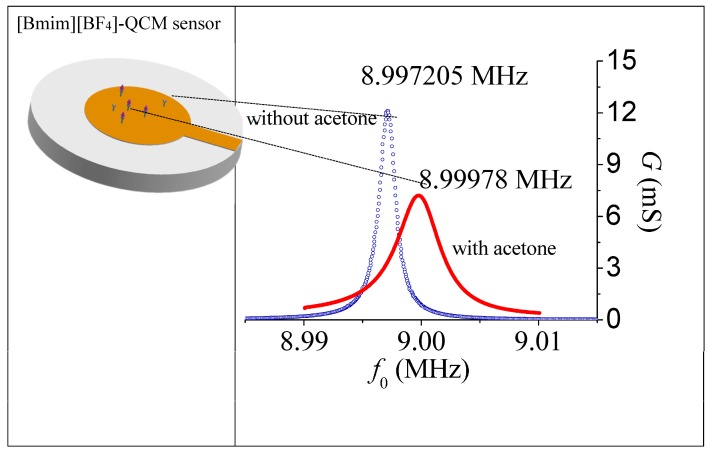
Detection of acetone vapor on the [Bmim]BF_4_-quartz crystal microbalance (QCM) sensor was monitored using the piezoelectric quartz crystal impedance (PQCI) technique.

The frequency response was measured using a network analyzer (E5016B, Agilent Technologies, Santa Clara, CA, USA). Since [bmim][BF_4_] would be used in ambient conditions, the [bmim][BF_4_]-modified QCM sensor was sealed in a glass chamber and equilibrated with air for 30 min at a stirring rate of 1500 rpm before measurement. All measurements were carried out at stable room temperature (22 ± 1 °C). Adsorption of acetone vapor was monitored using piezoelectric quartz crystal impedance measurements on the [Bmim]BF_4_-QCM sensor. The detection principle is shown in Scheme 1.

## 3. Results and Discussion

### 3.1. Viscoelasticity Effect of [bmim][BF_4_] Film

Most films behave neither as a rigid layer nor as an ideally viscous (Newtonian) membrane and therefore have to be treated as viscoelastic films [30]. The viscoelasticity of a film is evaluated by measuring the conductance using the PQCI technique [31]. Therefore, we investigated in detail if there was any difference in half bandwidth of the conductance spectra between the bare and modified electrodes. The conductance (*G*) at half peak height and half bandwidth of the conductance spectrum $\Delta f_{G_{1/2}}$ can be calculated as [32]:

(4) $$G=\frac{G_{\max}}{2}=\frac{1}{2R_{1}}$$

(5) $$\Delta f_{G_{1/2}}=f_{HG_{1/2}}-f_{LG_{1/2}}=\frac{R_{1}}{2\pi L_{1}}$$

(6) $$Q=\frac{f_{0}}{\Delta f_{G_{1/2}}}$$

where $f_{HG_{1/2}}$ and $f_{LG_{1/2}}$ are the higher and lower frequency at half peak height (*G* = *G_max_*/2) in the conductance spectrum, respectively; and *R*_1_ and *L*_1_ are the motional resistance and the motional inductance, respectively. *f*_0_ is the resonant frequency and *Q* is the quality factor. 

**Figure 1:**
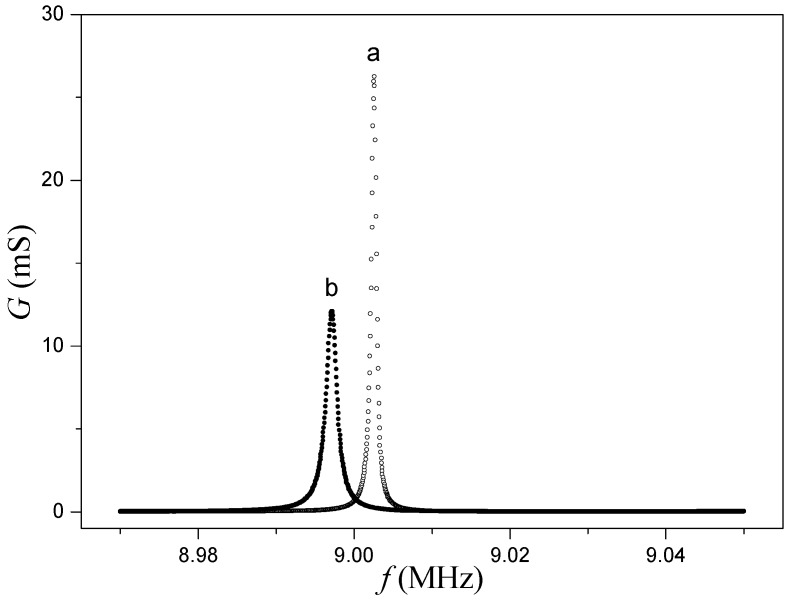
Effect of the presence of the [bmim][BF_4_] film on the conductance spectrum of quartz crystal in air: (**a**) bare QCM; (**b**) [bmim][BF_4_] film-modified QCM.

Figure 1 shows the conductance spectrum for the bare and modified electrodes. The calculated *G_max_* and $\Delta f_{G_{1/2}}$ values are given in Table 1. In Figure 1, plots a and b show the conductance spectrum corresponding to the bare quartz crystal and the [bmim][BF_4_] modified piezoelectric quartz crystal in air, respectively. Appreciable change in both peak height and half bandwidth of the conductance spectrum can be found. It can be concluded that the [bmim][BF_4_] film is nearly viscoelastic, not rigid, which agrees with the report [26].

The absolute value of Δ*f*_0_/Δ*R*_1_ is conventionally used to reflect whether frequency shift is dominated by mass loading or by the viscosity-density effect of the solution near the interface. If the value is more than 10 Hz/Ω for 9 MHz quartz crystal, the frequency shift is dominated by mass loading [33]. Otherwise, the frequency shift is dominated by the viscosity-density effect of the solution near the interface. From Table 1, the absolute value of Δ*f*_0_/Δ*R*_1_ is 123 Hz/Ω, much greater than 10 Hz/Ω. This indicates that the frequency shift due to the deposition of the [bmim][BF_4_] film on the QCM device is dominated by mass loading, and the viscosity-density effect of the solution near the interface can be negligible.

### 3.2. Response Curve on the [bmim][BF_4_]-Modified QCM Sensor

**Figure 2:**
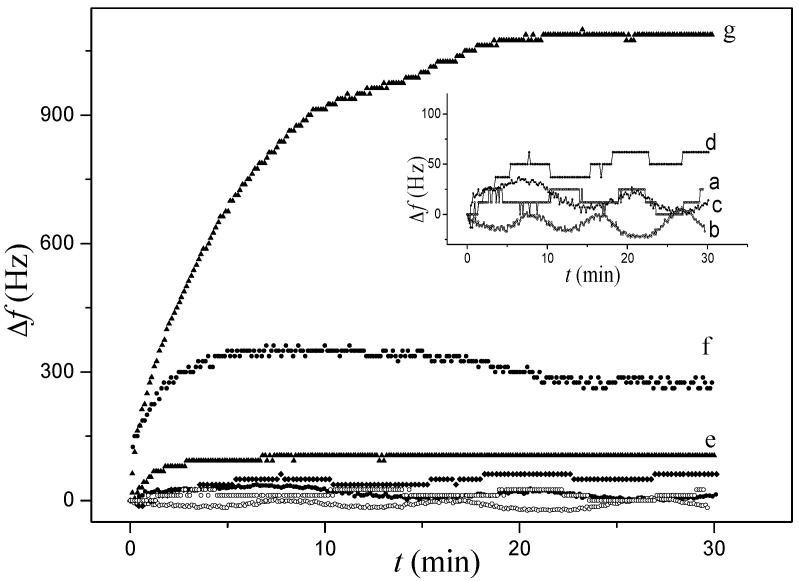
Typical [bmim][BF_4_]-modified QCM sensor response curves to different concentrations of acetone vapor: (**a**) 0; (**b**) 5.64; (**c**) 7.05; (**d**) 9.40; (**e**) 28.20; (**f**) 65.80; and (**g**) 329 ppmv. The inset figure is the enlargement of the curves at the bottom.

Commonly, there are two types of sample handling systems: dynamic and static. In this work, the static system was adopted. The modified-QCM sensor was placed in a sealed glass chamber with a specific volume. A typical [bmim][BF_4_]-modified QCM sensor response curve under exposure to different acetone concentrations is shown in Figure 2. Noticeably, the resonance frequency of the [bmim][BF_4_]-modified QCM device increased sharply when in contact with acetone vapor. The response time was measured at less than 10 s. The positive response, whose value is 1355 Hz for 329 ppmv acetone, is opposite to that of conventional QCM devices based on the Sauerbrey equation. If all 329 ppmv acetone were adsorbed on a QCM sensor coated with a rigid film, the corresponding negative frequency shift would be less than 13.3 Hz based on the Sauerbrey equation. The principle is based on the detection of mass loading, where the adsorption of gas to a rigid (or solid) polymeric or metallic film decreased the resonance frequency of the quartz crystal. Since the [bmim][BF_4_] film has been shown to be nearly viscoelastic, not rigid, we cannot use the Sauerbrey equation to explain our results.

The frequency shift of the viscoelastic film-modified QCM sensor results from the change in the physicochemical properties of the surrounding medium primarily is attributed to the film density and viscosity. The dependence of frequency shift (Δ*f*) on medium density and viscosity can be derived from Kanazawa and Gordon’s work [34] and is shown below:

(7) $$\Delta f=-f_{0}^{\frac{3}{2}}\sqrt{\frac{\rho_{L}\eta_{L}}{\pi \rho_{Q}\mu_{Q}}}$$

where *f*_0_ is the resonance frequency of the bare QCM sensor, and *ρ_L_* and *η_L_* are the absolute density and viscosity of the medium (or the film), respectively. *µ_Q_* and *ρ_Q_* are the elastic modulus and density of quartz, respectively. The solubility of organic vapor in imidazolium-based RTILs are known to be higher as compared with those of polymers [26]. The dissolution of organic vapor in RTILs can result in changes to its physicochemical properties [34,35,36,37]. In this work, adsorption of acetone vapor onto [bmim][BF_4_] film caused a reduction in viscosity and density of the film. The viscosity for pure acetone and [bmim][BF_4_] are 0.31 and 136.90 mPa·s, respectively. For the RTILs system, the reduction of viscosity is more pronounced than that of density after the addition of low-viscosity organic compounds [37,38]. The reason may be that entrapped low-viscosity organic compounds decrease ion-pairing or ion aggregation of RTILs by solvating interacting anions and cations. Therefore, the observed large and positive frequency shift (1355 Hz) must be related to a decrease in viscosity and density of the [bmim][BF_4_] film upon adsorption of acetone vapor. 

### 3.3. Calibration Curve

**Figure 3:**
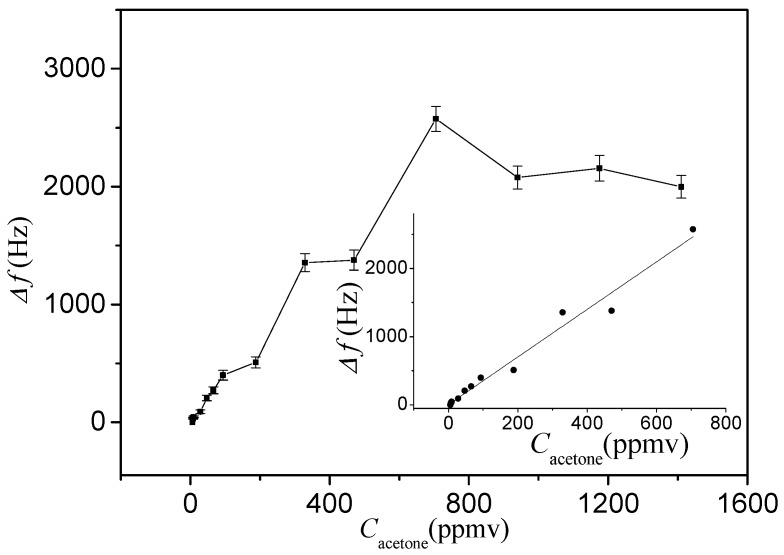
The relationship between the response frequency shift and acetone concentration on the [bmim][BF_4_]-modified QCM sensor. The inset figure is the calibration curve of acetone.

Figure 3 shows measurements of different acetone concentrations ranging from 0 to 1410 ppmv. A linear response range from 7.05 ppmv to 705 ppmv was found. The regression equation is: Δ*f* (Hz) = 3.49*C_acetone_* (ppmv) + 5.29, with a correlation coefficient of 0.990. The slope of this linear zone is 3.49 Hz/ppmv, which could be viewed as the sensitivity of the [bmim][BF_4_] modified-QCM sensor. The limit of detection is 5.0 ppmv, which is much lower than 77.6 ppmv and a little higher than 1.2 ppmv reported in previous works [39,40]. We define LOD as the lowest acetone concentration in air that can be measured with a signal to noise ratio of 3:1. For diabetic patients under different conditions, mean acetone concentration is found from 2.05 to 5.58 ppmv, while in healthy individuals, the value is in the range of 0.48 to 0.51 ppmv. Therefore in a real situation, a 10-fold pre-concentration value is needed. Meanwhile, the large response sensitivity is higher than previously reported values, such as 8.58 × 10^−3^ Hz/ppmv [26] and 0.52 Hz/ppmv [27].

### 3.4. Effect of Humidity on the [bmim][BF_4_]-Modified QCM Sensor

**Figure 4:**
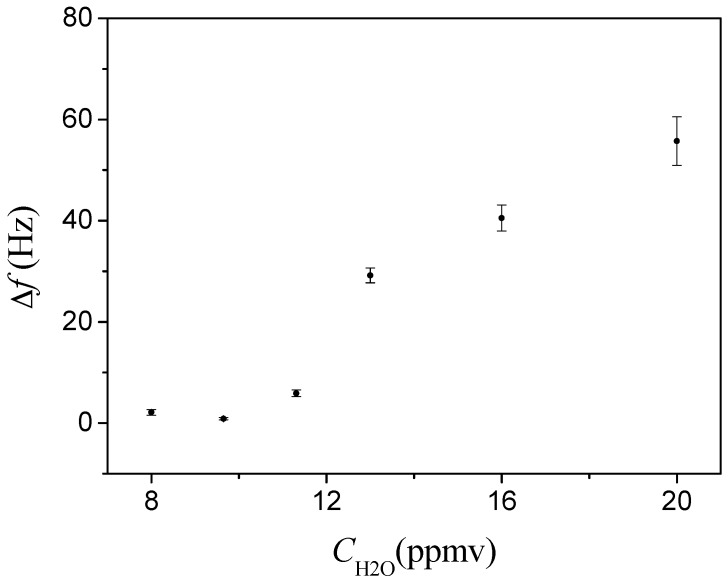
The effect of humidity on the [bmim][BF_4_]-modified QCM sensor.

Water vapor is a common interference to most sensors, and is especially important for gas sensors. Since the saturated water vapor concentration is 19.3 ppmv at 22 °C, we care about water vapor concentrations lower than this value. The effect of humidity on the [bmim][BF_4_]-modified QCM sensor was investigated and the results are shown in Figure 4. There is almost no frequency shift when water vapor concentrations were below 9.65 ppmv. Above 9.65 ppmv, there were positive frequency shifts of 5.9 Hz at 11.31 ppmv and 40.5 Hz at 16 ppmv. These results suggest that the sensing capability of the [bmim][BF_4_]-modified QCM sensor is slightly affected at high humidity conditions.

In this work, all measurements were performed with a relative humidity of 50% to 60% at a stable room temperature of 22 ± 1 °C. It means that the highest water vapor concentration did not exceed 11.58 ppmv. Therefore, the reported results do not need to be corrected. The effect of humidity will be considered for future iterations under highly humid environments, such as exhaled gas in human breath. In that case, the background response will be measured and subtracted for more accurate sensing results.

In real sample testing, there are at least two efficient routes to decrease the water vapor concentration. One is to pre-concentrate sample through the drying unit. Another route to address this issue is to entrap a useful additive into the RTIL sensing layer, such as chromium (III) based MIL-101, which is reported in Ref. [41].

### 3.5. Selectivity

**Figure 5:**
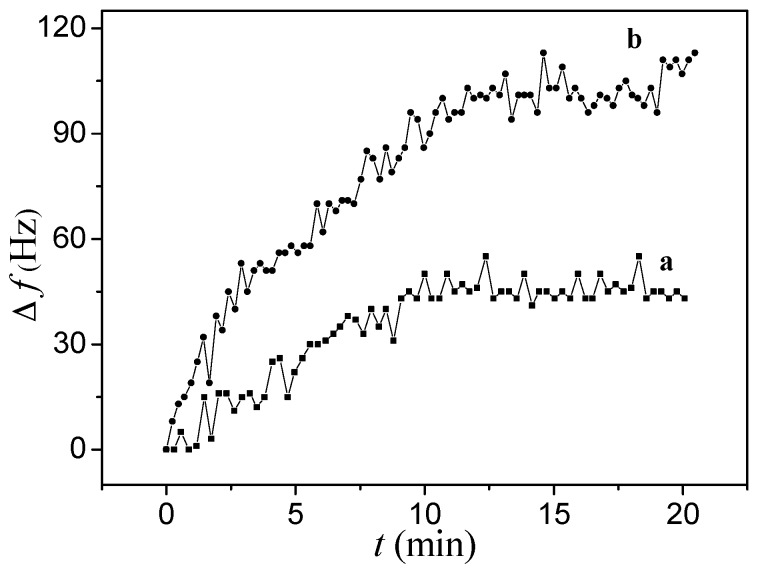
The response of the [bmim]BF_4_-modified QCM sensor to mixed VOC gases in the absence of acetone (**a**) and the presence of 9.25 ppmv acetone (**b**).

The most abundant VOCs with positive alveolar gradients in human breath are as follows: isoprene (48.60%), 1,2-pentadiene (15.00%), acetone (14.59%), *d*-limonene (8.43%), and *dl*-limonene (2.31%) [42]*.* In Table 2, a small response was observed in the three VOCs: 1,2-pentadiene, *d*-limonene and *dl*-limonene. The response values in Table 2 were comparable to background noise (around 13.0 Hz). However, the response to isoprene (18.1 Hz) is a little bit higher than the background noise, which is not comparable to that of 9.25 ppmv acetone (38.1 Hz). We also determined 9.25 ppmv of acetone in the presence of mixed VOC gases with comparable concentrations: isoprene (29.98 ppmv), 1,2-pentadiene (9.25 ppmv), *d*-limonene (5.2 ppmv), and *dl-*limonene (1.43 ppmv). The response of the [bmim]BF_4_-modified QCM sensor to mixed VOC gases in the absence of acetone were also tested as a control. Results are shown in Figure 5. There is a 104.0 Hz response for acetone in the presence of mixed VOC gases and 46.0 Hz for only mixed VOC gases. Therefore, we can determine acetone in the mixed VOC gases. This demonstrates that the [bmim][BF_4_] film presents anti-interference ability to the four VOCs to some extent. The reason may be that: the viscosity of isoprene, 1,2-pentadiene, *d*-limonene, and *dl*-limonene are 0.23, 0.25, 0.92, and 0.88 mPa·s, respectively. Only limonene has a higher viscosity value than that of acetone (0.31 mPa·s). Meanwhile, there is a carbonyl group on acetone molecules (CH_3_COCH_3_), while there is no such functional group or oxygen atom in the other VOCs (C_5_H_8_, C_10_H_20_). So the other organic interferents are non-polarizable molecules, while acetone is. The [bmim][BF_4_] film is a polarizable compound, which prefers binding with partially polarized acetone according to the principle that similarly polarized substances are more likely to be dissolved by each other. Therefore, the decrease of viscosity would be more pronounced for the acetone-[bmim][BF_4_] system than for those of the other four interferents-[bmim][BF_4_] systems. The frequency shift would be larger for acetone response on the [bmim][BF_4_]-modified QCM sensor.

### 3.6. Comparison of QCM and Gas Chromatography Measurements

**Figure 6:**
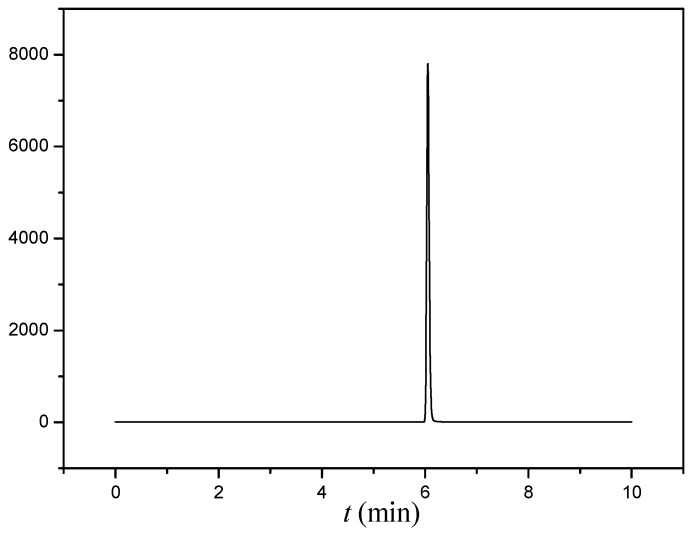
Chromatogram of 470 ppmv acetone in air.

Figure 6 shows the chromatograms of 470 ppmv acetone. An obvious peak was observed in the chromatograms at a retention time of 6.05 min. The same sample was also added into the gas chamber, and then tested by the [bmim][BF_4_]-modified QCM sensor. The response frequency shift was about 1425 Hz. From the regression equation, the acetone concentration obtained from the QCM method was about 407 ppmv. This result is comparable to the value determined by the GC technique. Therefore, the QCM method can be a complementary method to the GC technique. In addition, the presence of water vapor is an unfavorable factor in the detection of VOCs by the GC technique, while the [bmim][BF_4_]-modified QCM sensor is not subject to this restriction; it allows trace water to be present. 

## 4. Conclusions

A 9 MHz QCM sensor has been successfully developed, evaluated, and tested for acetone vapor employing room temperature ionic liquids ([bmim][BF_4_]) as sensing materials. The [bmim][BF_4_] film has been proven to be viscoelastic, which relates the response of the modified QCM sensor to the viscosity and density change of the film. The [bmim][BF_4_] modified-QCM sensor demonstrated a linear range from 7.5 to 705 ppmv, with a fast response to acetone vapors because of the fast diffusion of analytes in RTILs. Our preliminary results will stimulate further research towards developing noninvasive, inexpensive, portable, and compact sensors for early diabetes diagnosis.

## Figures and Tables

**Table 1:** Comparison of bare and modified QCM in air by PQCI.

Items	*G*_max_ (mS)	$\Delta f_{G_{1/2}}$ (Hz)	*Q*	Δ*R*_1_ (Ω)	Δ*f*_0_/Δ*R*_1_ (Hz/Ω)
Bare QCM	26.30	750	12,004	NA	NA
[bmin][BF_4_]-modified QCM	12.08	1700	5293	44.36	123

NA: not applicable.

**Table 2:** Response of [bmim][BF_4_]-modified QCM sensor to four common interferents.

Interferents	*C* (ppmv)	Δ*f* (Hz)
Isoprene	29.98	18.1
1,2-pentadiene	9.25	12.5
*d*-limonene	5.20	11.7
*dl*-limonene	1.43	13.4
